# Assessing the impact of generative AI on undergraduate thesis quality: A comparative study of students and teachers

**DOI:** 10.1371/journal.pone.0347653

**Published:** 2026-04-28

**Authors:** Guangjian Yan, Chenhao Zhu, Tong Li, Jinchi Yip, Jing Ni

**Affiliations:** 1 Art School, Anqing Normal University, Anqing, Anhui, China; 2 Design School, Taylor’s University, Sunway, Selangor, Malaysia; Institute of Medical Biochemistry Leopoldo de Meis (IBqM) - Federal University of Rio de Janeiro (UFRJ), BRAZIL

## Abstract

Generative AI (GenAI) is increasingly embedded in undergraduate thesis work, intensifying concerns about thesis quality. However, limited evidence is available on how GenAI engagement relates to teachers’ and students’ evaluations of thesis quality and whether these associations differ across institutional and disciplinary contexts. Using a stratified random sampling method across institutional tiers and disciplines, 934 participants were recruited (684 graduating students and 250 thesis teachers). Key variables (Extent of GenAI Involvement (EX), Perceived Effect on Thesis Quality (EF), Perceived Problems/Risks (PR), Attitudes Toward GenAI Use (AT), Perceived Writing Ability Development (AB)) were measured using structured scales, then five-step hierarchical regression analysis was employed to estimate main effects and test interactions. Results showed that EX (*B* = 0.200, *p* < .001) and AB (*B* = 0.185, *p* < .001) were positively associated with EF; AT showed a marginal association (*B* = 0.037, *p* = .053) and policy presence showed a small positive association (*B* = 0.118, *p* = .001). EX/AB/PR/AT and Group interactions increased explanatory power (*R*^2^ = .445; $\Delta R^{2}=\mathrm{.081}$), PR was not significant (*p* = .220). Policy did not moderate Group differences ($\Delta R^{2}=\mathrm{.000}$, *p* = .684). Institutional tier and Group interactions further improved fit ($\Delta R^{2}=\mathrm{.035}$), strongest in World-Class Universities (*B* = 0.986, *p* < .001). Disciplinary-category and Group interactions added incremental variance ($\Delta R^{2}=\mathrm{.030}$; final *R*^2^ = .510), with the largest teacher–student gap in Natural Sciences. The findings revealed that EF was most consistently linked to EX and AB, with systematic heterogeneity by group and by institutional and disciplinary context, underscoring the need for differentiated guidance on policy-compliant, capability-oriented GenAI use; however, given the cross-sectional and self-reported design, EF captures perceived thesis quality.

## 1 Introduction

### 1.1 Research background

Undergraduate theses are both a comprehensive demonstration of students’ academic abilities and research training, and a key lens for observing the quality of university teaching. In recent years, Generative Artificial Intelligence (GenAI) has entered multiple stages of the thesis process; with its “low barrier to entry, rapid feedback, and broad coverage across tasks,” it can lower the costs of expression and information integration, yet simultaneously introduces new challenges for originality, academic integrity, and traceability [1–3]. In late 2024, the release of DeepSeek further accelerated the uptake of GenAI tools in Chinese higher education and expanded the practical availability of advanced assistants for thesis writing [4–6]. In the context of Chinese higher education, university governance of GenAI remains heterogeneous: some institutions have issued usage guidelines and risk-screening procedures, while others are still experimenting or leaving gatekeeping to schools or individual advisers; moreover, boundaries for acceptable use vary between disciplines and institutional tiers.

The combined shifts in tool usability, institutional norms, and assessment orientations are reshaping the practical ecology of how students write and how instructors evaluate. However, to what extent GenAI is associated with thesis-quality evaluations—and whether such associations differ across institutional tiers, disciplinary paradigms, and student–teacher perspectives—remains insufficiently evidenced within a unified quantitative framework.

### 1.2 Literature review

#### 1.2.1 GenAI involvement in undergraduate thesis work.

Due to their coherent text generation and multimodal information-integration capabilities, AI systems have become accelerators of educational innovation, reshaping aspects of teaching and learning [2,7]. With large-scale pretraining and substantial computational power, GenAI can address questions across disciplines and produce long-form text resembling human writing—uses that some degree applicants have leveraged for thesis drafting or even ghostwriting [8]. “AI ghostwriting” is not limited to generating a whole thesis; it also includes piecemeal text generation, paraphrasing, and attempts to reduce similarity indices through rewriting [9]. These diverse practices suggest that GenAI use varies in intensity and task coverage rather than being a simple yes/no choice, motivating the construct of the extent of GenAI involvement in thesis work.

#### 1.2.2 Benefits and risks of GenAI use.

Existing studies broadly follow two lines. One body of evidence highlights positive effects: GenAI can improve linguistic quality and textual coherence, support outline generation, information integration, and paragraph rewriting [10], thereby enhancing writing efficiency and expression and easing the burden of initial drafting [11]. A second line foregrounds risks—over-reliance, hallucinations and factual errors, citation improprieties, insufficient methodological traceability, and diluted originality—especially when usage is not accompanied by labeling, process logs, and verification procedures [12]; under such conditions, the boundary between tool output and learners’ own academic competence can blur [13]. Taken together, these two lines imply two complementary mechanisms for how GenAI relates to thesis-quality evaluations: a competence-building pathway reflected in perceived writing ability development, and a risk-appraisal pathway reflected in perceived problems/risks, both of which can shape perceived effects on thesis quality.

#### 1.2.3 Contextual and stakeholder heterogeneity.

These impacts of GenAI are also context-dependent: disciplinary differences are pronounced, with humanities work often prioritizing semantic quality in content generation and engineering emphasizing efficiency in data analysis and the write-up of experimental results [14]. At the institutional level, governance remains heterogeneous; universities’ GenAI policies vary in scope and emphasis, shaping how “being able to use” translates into “using well” [15,16]. Finally, perceptions diverge across actors: students tend to register immediate gains in language/structure and efficiency, whereas instructors concentrate on compliance and demonstrable contributions across stages, leading to different criteria for judging “quality” [17]. Such role-based differences suggest that attitudes toward GenAI use may not map onto quality evaluations in a uniform way and should be interpreted alongside GenAI participation in writing ability, problem and risk, rather than as a standalone explanation

#### 1.2.4 The current study.

Taken together, existing work offers helpful but fragmented evidence on GenAI in academic writing and thesis work. Definitions and operationalizations vary between studies—especially for “thesis quality,” which can refer to surface-level fluency and coherence in some research but integrity, traceability, and demonstrable competence in others—reducing the comparability between studies. Moreover, many studies rely on broad indicators of GenAI use or general attitudes, which obscure distinct underlying processes and makes it difficult to differentiate scaffolded support from substitutional practices (e.g., ghostwriting or concealment-oriented rewriting). Finally, stakeholder and contextual heterogeneity (e.g., teacher–student roles, institutional governance arrangements, and disciplinary norms) is frequently discussed descriptively rather than jointly tested within a unified framework.

Consequently, existing evidence does not yet specify the mechanisms through which GenAI is linked to thesis-quality evaluations, nor the conditions under which such evaluations diverge across stakeholder roles and contexts, leaving unclear when and for whom GenAI is perceived as beneficial in undergraduate thesis work.

### 1.3 Problem statement and research questions

Despite valuable prior work, a comprehensive evaluation of GAI in undergraduate thesis quality remains limited, prior work leaves three actionable gaps: (i) a unified test of four individual-level factors—participation, ability development, rule-compliant use, and attitudes—under a dual-pathway view; (ii) a like-for-like comparison between teachers and students; and (iii) boundary conditions shaped by university tier, internal policy, and disciplines. Within China’s higher-education governance context, there is still a lack of systematic comparisons of how institutional tier and the enforcement of internal policies alter these relationships

For conceptual clarity, this study focuses on five constructs. Extent of GenAI involvement (EX) refers to how intensively and broadly GenAI is used across thesis tasks and stages. Perceived writing-ability development (AB) captures stakeholders’ perceptions that GenAI use contributes to the development of writing- and research-related competence. Perceived problems/risks (PR) reflect integrity- and reliability-related concerns such as hallucinations, citation improprieties, diminished traceability, over-reliance, and diluted originality. Attitudes toward GenAI use (AT) denote an overall motivational orientation toward adopting GenAI in thesis work. Perceived effect on thesis quality (EF) represents stakeholders’ evaluations of how GenAI use relates to thesis quality, and is treated as a perceived outcome rather than an objectively graded score. Based on this, we pose the following research questions:

- RQ1: How are individual-level factors—GenAI participation extent, ability development, perceived risk and problem, institutional GenAI policies and attitudes—associated with perceived thesis quality?
- RQ2: Do these associations differ between teachers and students?
- RQ3: Do the university tier and the presence of an explicit institutional policy relate to evaluations and/or group differences?
- RQ4: Do disciplines exhibit systematic differences in adjusted evaluations of thesis quality?

## 2 Methods

### 2.1 Research design

This study employed an exploratory sequential mixed-methods design (QUAL→QUAN) with a building integration logic and a QUAN emphasis. Phase 1 themes were used to refine construct boundaries and wording, generate and revise items, and identify salient heterogeneity to be tested via interaction terms in Phase 2.

Phase 1 (QUAL: construct and item development). Guided by the literature review, the study first articulated a construct map that distinguished a competence-building pathway (EX, AB) and a risk-appraisal pathway (PR), with AT capturing motivational orientation toward GenAI and EF representing thesis-quality evaluations. Then semi-structured interviews were conducted with current graduating cohorts and their thesis advisors. Interviews focused on (a) typical thesis-related activities where GenAI is used, (b) perceived benefits and risks/problems, and (c) criteria stakeholders apply when judging thesis quality. Using thematic analysis, we derived a codebook of mechanisms and language cues. These insights informed the questionnaire development process, ultimately guiding the generation and refinement of items and constructs.

Phase 2 (QUAN: survey and quantitative analysis). A large-scale survey was administered under a stratified framework by institutional tier × disciplinary category, comparing students and teachers as two distinct groups to examine differences in GenAI use and evaluations of thesis quality and to obtain differentiated quantitative results.

### 2.2 Participants and sampling

The target population comprised the 2025 graduating cohort of undergraduates and their thesis advisors in mainland China. To enhance coverage and breadth, the sample covered multiple institutional tiers—world-class universities, first-class discipline universities, provincial key (Category I) universities, and local non-key institutions—following the official classification criteria for the current round of “Double First-Class” construction [18]. To facilitate understanding for readers from other countries, we have used the term “world-class” to refer to the corresponding concept of the first classification. “World-Class University” refers to universities designated for world-class university construction; “First-Class Discipline” refers to institutions with designated world-class discipline(s) (but not in the world-class university construction list). “Provincial Key” denotes universities formally identified as key institutions at the provincial level, and “Ordinary (non-key)” denotes local institutions not included in the above categories. Disciplinary fields followed the Fields of Science and Technology classification, including Natural Sciences, Engineering & Technology, Medical & Health Sciences, Agricultural Sciences, Humanities, Social Sciences, and Interdisciplinary Studies [19,20]. A stratified sampling framework was employed to ensure adequate coverage and cell sizes for subgroup comparisons [21].

Recruitment followed a two-stage procedure. At the institutional level, eligible universities were identified from official national lists and stratified by institutional level and disciplinary distribution; within each stratum, institutions were randomly selected using a computer-generated procedure and contacted sequentially until the target quota for the stratum was reached. After an institution agreed to participate, relevant programs/fields were approached to match the predefined disciplinary categories, and the survey link/QR code was disseminated to eligible final-year undergraduates and their thesis advisors through online and on-site channels.

This study was approved by the Ethics Committee of the Scientific Research Office, Anqing Normal University (Approval No. ANU2024102), and was conducted in accordance with institutional ethical requirements. Participation was voluntary; all participants provided informed consent and could withdraw at any time without penalty. All data were anonymized and treated confidentially.

### 2.3 Data collection and analysis

#### 2.3.1 Instrument development.

The development of the questionnaire followed a literature-informed and interview-driven process to ensure both theoretical grounding and contextual relevance.

Construct identification. A comprehensive review of literature on undergraduate thesis quality [22] and generative AI (GenAI) in academic writing was first conducted to delineate potential dimensions of impact, focusing on participation, ability development, perceived risks and problems, attitudes, and perceived thesis-quality outcomes [23–25]. These conceptual domains were aligned with the “competence-building” and “risk-appraisal” pathways established in prior studies.

Semi-structured interviews. To complement the literature and capture context-specific language and experiences, semi-structured interviews were conducted with teachers (n = 12) and students (n = 18) across different university tiers and disciplinary fields. The interview guide explored (a) stages of the thesis process where GenAI was used, (b) perceived benefits and challenges, and (c) individual criteria for judging thesis quality.

Coding reliability. Interview transcripts were imported into NVivo 14 for thematic analysis. An initial codebook informed by the interview guide and the literature was developed and then refined it iteratively through inductive coding [26]. Two researchers independently coded the transcripts in NVivo using this evolving codebook. Coding differences were reviewed in regular consensus meetings, during which code definitions and boundaries were clarified and the codebook was updated accordingly; the revised codebook was then applied in subsequent coding cycles. Throughout the process, analytic memos and versioned records of codebook revisions were maintained as an audit trail to enhance transparency and dependability [27]. In addition, the research team conducted peer debriefing when finalizing themes into candidate questionnaire items to strengthen interpretive credibility of content relevant to GenAI use in thesis work [28].

Item generation and refinement. Insights from the literature and interviews were integrated into an initial item pool representing five constructs: GenAI participation, ability development, perceived risks and problems, attitudes toward GenAI, and thesis-quality evaluations. Redundant or overlapping items were removed, and parallel wording was developed for teacher and student versions to enhance comparability.

Expert validation. Three subject-matter experts (two in education and one in computer science) independently reviewed all items for content relevance, clarity, and alignment with construct definitions using the Content Validity Index (CVI) procedure [29,30]. Items meeting accepted CVI thresholds were retained and refined for clarity and sequencing.

Final instrument structure. The finalized questionnaire adopted a 7-point Likert scale (1 = strongly disagree, 7 = strongly agree) and was organized into six coherent sections: (1) Background information, including institutional tier, disciplinary field, gender, and the presence of an institutional GenAI policy; (2) Extent of GenAI involvement by thesis section (EX), capturing how frequently and deeply GenAI was used across stages of the thesis process; (3) Perceived effect on thesis quality (EF); (4) Perceived problems and risks after GenAI use (PR); (5) Attitudes toward GenAI use in thesis writing (AT); (6) Perceived impact on writing ability development (AB). This structure ensured that the instrument covered both contextual characteristics and the five focal constructs examined in this study.

This process ensured that the final instrument was both theoretically grounded in existing standards and empirically informed by real experiences of GenAI use in the thesis-writing process, thereby achieving strong content validity and contextual fit for the subsequent quantitative analysis.

#### 2.3.2 Survey administration and pilot item analysis.

The questionnaire was distributed online via Wenjuanxing to both 2025 undergraduate cohorts and their thesis advisors. Before the main survey, a pilot test was conducted with students (n = 61) and teachers (n = 32) to examine wording, flow,and item performance [31]. Following standard extreme-groups item analysis, respondents were split by total score into the top 27% and bottom 27%, and independent-samples t tests were used to assess item discrimination. Results indicated significant between-group differences for all items (*p* < .05), suggesting satisfactory discrimination; therefore, all items were retained for the full survey [32,33]

#### 2.3.3 Data analysis.

Data preprocessing. To make scales comparable and reduce multicollinearity introduced by interaction terms, all continuous predictors were standardized and mean-centered prior to modeling. Specifically, EX, AB, PR, and AT were converted to z scores before entering the regressions. To facilitate interpretation of the intercept and to mitigate multicollinearity in interactions with binary variables, binary grouping variables were also mean-centered: identity (student vs. teacher) was centered and denoted GrC, and existence of institutional AI policy was centered and denoted UrC. On this basis, we constructed interaction terms between the core predictors and group (e.g., EX×GrC, AB×GrC, AT×GrC, RU×GrC), as well as the interaction between institutional policy and group (UrC×GrC).

Categorical variables and dummy coding. University Tier (UT; 4 groups) and Disciplinary field (DT; 7 groups) entered the models via dummy coding. For interpretability, local non-key universities and Natural Sciences served as the reference groups. Accordingly, UT was represented by three dummies—UT2 (provincial key universities), UT3 (First-Class Discipline universities), UT4 (Double First-Class universities)—each contrasted with the reference tier. DT was represented by six dummies—DT2 (Engineering and Technology), DT3 (Medical and Health Sciences), DT4 (Agricultural Sciences), DT5 (Social Sciences), DT6 (Humanities), DT7 (Interdisciplinary Studies)—each contrasted with Natural Sciences. All interactions with Group were computed by multiplying the above dummies by GrC.

Model selection. Hierarchical regression was used to evaluate the incremental explanatory contribution of conceptually defined predictor blocks to EF, and to test boundary conditions via interaction terms. Model entry followed the research questions: control variables were entered first, followed by individual-level predictors (EX, AB, RU, AT) to estimate their unique associations with EF; Group (teacher vs. student) interaction terms were then added to examine whether these associations differed between teachers and students; finally, institutional and disciplinary context terms and their interactions with Group were entered to evaluate contextual boundary conditions. Model improvement was assessed using $\Delta R^{2}$ and F-change statistics, and multicollinearity diagnostics (VIF) were examined to ensure stable estimation. Incremental effect sizes for each added block were computed as Cohen’s *f*^2^


$$\Delta f^{2}=\frac{\Delta R^{2}}{1-R_{\text{new}}^{2}}$$

Where $R_{\text{new}}^{2}$ is the $\Delta R^{2}$ of the model after adding the block at that step [34].

## 3 Result

### 3.1 Descriptive statistics of participants

Using both on-site and online channels, 1,028 participants completed the questionnaire. After applying pre-specified data-screening criteria [35], 94 submitted responses were excluded as invalid, yielding 934 valid cases for analysis (teachers n = 250; students n = 684). The valid-case rate was 90.86%. Invalid cases were defined as submitted questionnaires that met one or more exclusion criteria: (a) ineligible respondents outside the target population, (b) duplicate submissions, (c) failed attention checks, (d) straight-lining. By gender, 54.40% of teachers were male, and 45.60% were female; among students, 55.30% were male, and 44.70% were female. The teacher sample was dominated by associate professors (26.40%) and lecturers (25.6%); by institutional tier, local non-key universities (27.60%) accounted for the largest share. The student sample mainly came from first-class discipline (25.15%) and local non-key institutions (27.49%). In terms of disciplinary distribution, teacher respondents were concentrated in Humanities (17.20%), while student respondents were primarily from Humanities (15.93%) and Natural Sciences (14.77%). Frequencies and within-group percentages for all variables are reported in Table 1. Percentages are within-group proportions and may not sum to 100% due to rounding. Overall, the sample is balanced across institutional tiers and disciplinary categories, covering world-class universities, first-class discipline construction, provincial key, and local non-key institutions, as well as major fields such as Natural Sciences, Humanities and Social Sciences, and Interdisciplinary Studies, with no undue concentration in any single category.

**Table 1:** Sample Characteristics.

		Teacher		Student	
Variable	Category	No	%	No	%
Gender	Male	136	54.40	378	55.30
	Female	114	45.60	306	44.70
Academic Rank	Assistant	61	24.40	–	–
	Lecture	64	25.60	–	–
	Associate Professor	66	26.40	–	–
	Professor	59	23.60	–	–
Institutional Tier	World-Class University	65	26.00	156	22.81
	First-Class Discipline University	54	21.60	172	25.15
	Provincial Key University	62	24.80	168	24.56
	Local/Non-key Institution	69	27.60	188	27.49
Disciplinary Field	Natural Sciences	40	16.00	101	14.77
	Engineering & Technology	34	13.60	96	14.04
	Medical & Health Sciences	36	14.40	89	13.01
	Agricultural Sciences	36	14.40	98	14.32
	Social Sciences	31	12.40	92	13.45
	Humanities	43	17.20	109	15.93
	Interdisciplinary Studies	30	12.00	99	14.47

**Table Notes:** Percentages are reported as in the analysis output and rounded to two decimal places. Teacher group total *N* = 250. Student group total *N* = 684.

### 3.2 Reliability and validity of the questionnaire

Before the empirical analyses, we conducted factor analysis on the study variables. Using SPSS 27, the data showed KMO = .886 and a significant Bartlett’s test of sphericity (*p* < .001), indicating suitability for factor extraction according to common evaluation criteria [36]. Then principal components analysis was conducted to examine the underlying factor structure. Component retention was guided by eigenvalues >1, inspection of the scree plot, and the interpretability of the solution, with cumulative variance explained exceeding 50%. Items were screened iteratively and removed if they exhibited low primary loadings (< .40), substantial cross-loadings or low communalities, or if their removal was necessary to ensure that each retained component was supported by at least three items [37]. After deleting items that did not meet these criteria, a five-factor structure was obtained: EX (6 items), EF (6 items), PR (4 items), AT (4 items), AB (3 items), with primary loadings on their respective factors ranging from .56 to .85 (see Table 2). The total variance explained was 67.63%, which indicates acceptable structural validity by commonly used benchmarks [36,37].

**Table 2:** Exploratory Factor Analysis: Retained Items and Loadings.

Construct	Item (code & brief wording)	Factor Loading
Extent of GenAI Involvement by Thesis Section (EX)	EX4: Research methods	.847
	EX5: Data/content analysis	.837
	EX3: Literature Review	.835
	EX2: Introduction or research background	.829
	EX1: Abstract and keywords	.821
	EX6: Conclusion and discussion	.821
Perceived Effect on Thesis Quality (EF)	EF4: GAI improved the efficiency and quality of literature review and material analysis	.813
	EF5: GAI performed well in data analysis and in generating figures/tables	.790
	EF2: GAI tools had a positive effect on the thesis’s structure and logical coherence	.772
	EF6: GAI tools enhanced the overall quality of the thesis	.766
	EF3: GAI tools fostered innovation and novelty in thesis content	.716
	EF1: AI tools improved the thesis’s linguistic expression and grammatical accuracy	.588
Perceived Problems/Risks After GenAI Use (PR)	PR3: AI tools may reduce academic depth and originality of research	.855
	PR4: Direct use of AI-generated content in the thesis	.788
	PR2: AI-generated content may contain factual errors or misleading information	.772
	PR1: AI tool overreliance in thesis writing	.650
Attitudes Toward GenAI Use in Thesis Writing (AT)	AT4: GAI use in academic writing should be reasonably regulated and guided	.858
	AT5: Universities should issue clear policies on AI tools to guide thesis writing	.809
	AT3: GAI use needs to be explicitly disclosed in the thesis	.670
	AT2: Instructors should guide students in using AI tools for thesis work	.562
Perceived Impact on Writing Ability Development (AB)	AB2: GAI tools enhanced academic writing ability	.845
	AB3: GAI helps students understand and master academic writing conventions	.805
	AB1: GAI enhanced students’ problem-solving and independent thinking	.751

**Table note:** Loadings are standardized factor loadings. Items are listed under their primary factor.

In addition to the factor analysis and principal components analysis, the measurement properties of the five constructs (EX, EF, AB, RU, and AT) were evaluated by examining internal consistency (Cronbach’s α, composite reliability, CR), convergent validity (AVE), and discriminant validity using the Fornell–Larcker criterion [38]. Descriptive statistics (M, SD) were also reported to summarize overall response tendencies. As shown in Table 3, internal consistency indices met commonly accepted standards, AVE values supported convergent validity, and the square roots of AVE exceeded the inter-construct correlations, indicating adequate discriminant validity.

**Table 3:** Reliability, Convergent Validity and Discriminant Validity.

Construct	Reliability	Convergent Validity		Discriminant Validity					Descriptive Statistics	
	Cronbach’s α	CR	AVE	EX	EF	AB	PR	AT	M	SD
EX	.933	.878	.937	**.968**					4.85	1.13
EF	.835	.881	.554	.581	**.744**				5.02	0.89
AB	.762	.852	.593	.097	.326	**.770**			4.65	1.05
PR	.840	.82	.539	.334	.384	.258	**.734**		5.14	0.96
AT	.813	.843	.642	.325	.442	.168	.414	**.801**	5.67	0.85

**Table note:** Diagonal entries in bold are the square roots of AVE ($\sqrt{AVE}$); values in the lower triangle are inter-construct Pearson correlations.

### 3.3 Main effects and interaction effects

This study employed a five-step hierarchical regression to examine changes in explanatory power for the dependent variable EF from various main effects and their interactions with Group (teacher = 0, student = 1). The sample size was N = 934; VIFs < 5 across all models indicated no multicollinearity [39,40]. Model summaries are reported in Table 4, and coefficients for main and interaction terms are reported in Table 5

**Table 4:** Model Summary.

Model	R	R^2^	Adj.R^2^	SE	Δ*R*^2^	F-Change	df1	df2	p
1	.604	.365	.354	.515	.365	35.10	15	918	.000
2	.667	.445	.434	.482	.081	33.27	4	914	.000
3	.667	.445	.433	.482	.000	.16	1	913	.684
4	.693	.480	.467	.468	.035	20.19	3	910	.000
5	.714	.510	.494	.456	.030	9.20	6	904	.000

**Table 5:** Hierarchical regression predicting EF.

Model	Predictors	*B*	*Beta*	*t*	*p*	Lower	Upper	VIF
						CI	CI	
1	GroupC	.473	.327	8.962	.000	.370	.577	1.93
	URC	.118	.091	3.311	.001	.048	.189	1.09
	EX(z)	.200	.312	9.990	.000	.161	.239	1.41
	AB(z)	.185	.289	9.482	.000	.147	.223	1.34
	PR(z)	.101	.158	4.571	.000	.058	.144	1.72
	AT(z)	.037	.057	1.930	.053	−.001	.074	1.26
2	EX(z)×GrC	−.349	−.261	−8.490	.000	−.430	−.268	1.56
	AB(z)×GrC	.120	.089	3.180	.002	.046	.194	1.28
	PR(z)×GrC	.053	.038	1.241	.220	−.031	.137	1.54
	AT(z)×GrC	−.241	−.186	−6.580	.000	−.313	−.169	1.31
3	PRC×GrC	−.031	−.012	−.407	.684	−.181	.119	1.41
4	UT2×GrC	.453	.154	4.192	.000	.241	.665	2.37
	UT3×GrC	.359	.141	3.064	.002	.129	.589	3.71
	UT4×GrC	.986	.384	7.633	.000	.732	1.239	4.43
	DT2×GrC	−.623	−.151	−3.858	.000	−.941	−.306	2.82
5	DT3×GrC	.153	.035	.956	.339	−.161	.468	2.44
	DT4×GrC	−.059	−.018	−.412	.681	−.339	.222	3.53
	DT5×GrC	−.470	−.155	−3.366	.001	−.744	−.196	3.91
	DT6×GrC	−.483	−.190	−3.753	.000	−.736	−.231	4.72
	DT7×GrC	−.060	−.014	−.375	.708	−.372	.252	2.55

**Table note:** Predictors are z-standardized; Group coded 0 = Teacher, 1 = Student; UR and Group are mean-centered; UT reference = General universities; DT reference = Natural sciences; interaction terms formed by multiplying centered variables. CI refers to the 95% confidence interval (Lower and Upper bounds).

#### 3.3.1 Main effects.

Model 1 entered Institutional AI Policy, University Tier (dummy-coded), disciplinary category, Group, and the standardized GenAI predictors (EX, AB, RU, AT). The model explained 36.5% of the variance in EF (*R*^2^ = .365; Adj. *R*^2^ = .354). Controlling for other variables, students reported higher evaluations of AI’s impact on thesis quality (EF) than teachers (*B* = .473, *p* < .001). At the organizational level, the presence of an explicit policy (URC) showed a positive effect (*B* = .118, p *p* < .001). Among the four dimensions, EX was positively associated with EF (*B* = .200, *p* < .001), AB was positively associated with EF (*B* = .185, *p* < .001), PR was also positive (*B* = .101, *p* < .001), and AT was not significant (*B* = .037, *p* = .053)

#### 3.3.2 Main effects × group interactions.

**Fig 1:**
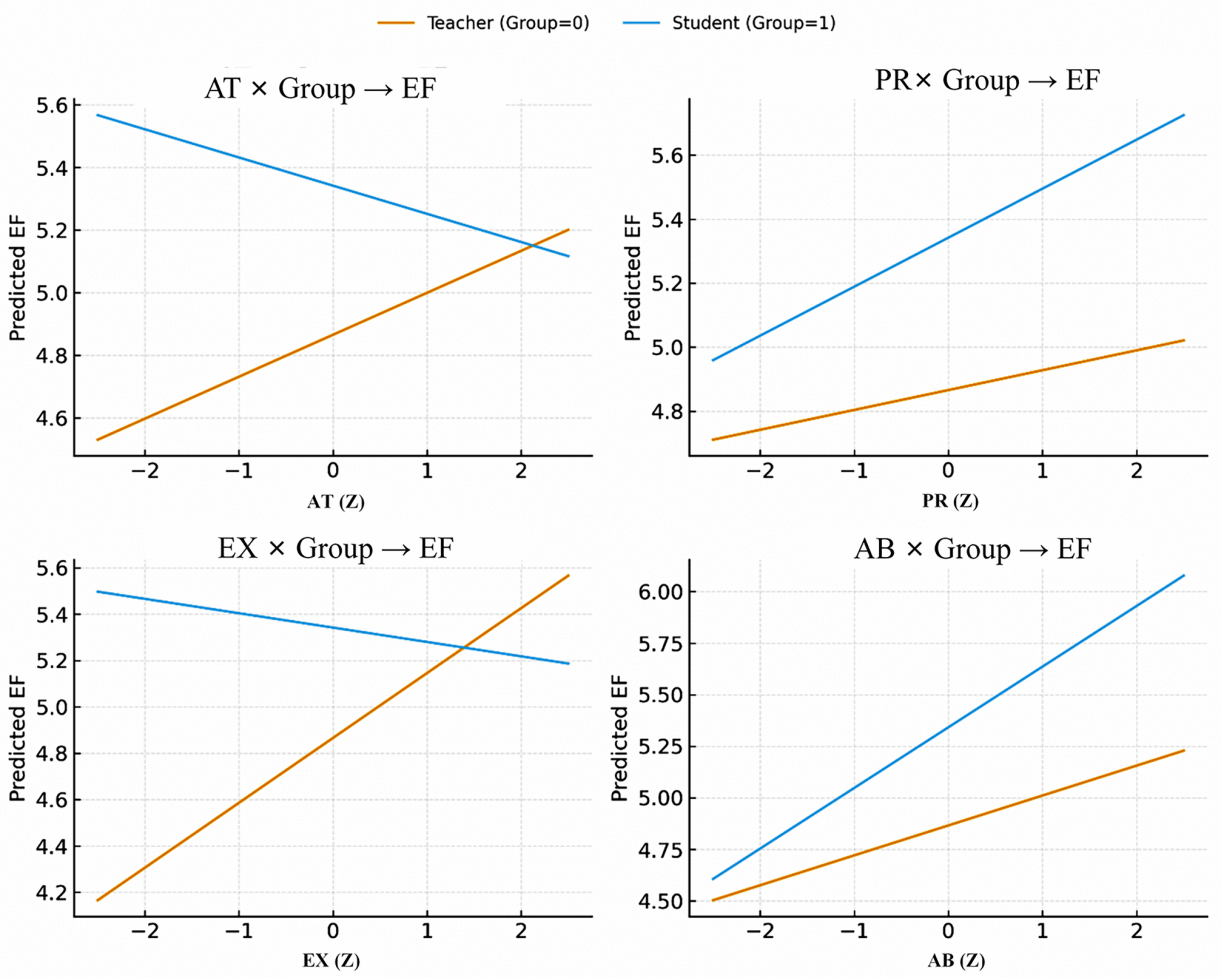
Simple slopes for the interactions between group and predictors on EF. Predicted EF is plotted across z-standardized AT, PR, EX, and AB for teachers and students, with other predictors held at *z* = 0. Panels show the interactions for AT, PR, EX, and AB. Teacher and student groups are represented by separate lines. AT × Group and EX × Group show stronger effects for teachers, AB × Group shows a stronger effect for students, whereas PR × Group shows a relatively weak interaction.

After adding the four interaction terms EX(z) × Grc, AB(z) × Grc, RU(z) × Grc, AT(z) × Grc, Model 2 reached *R*^2^ = .445, with adjusted *R*^2^ = .434; The interaction block yielded *f*^2^ = .146, indicating an approximately medium incremental effect. This indicates that Group significantly moderates the relationships of EX, AB, and AT with EF, whereas the RU × Group interaction is not significant. The directions of interaction are as follows (Table 5; The four simple-slope plots in Fig 1 provide visual support).

- EX × Group is negative (*B* = −.349, *p* < .001). With Group coded teacher = 0, student = 1, the teacher simple slope = *B*(EX) = + .200; the student simple slope = *B* (EX) + *B* (EX × Group) = −.149. This indicates that the positive effect of EX on EF is stronger for teachers, whereas it is substantially weakened, even turning negative, for students (see EX x Group of Fig 1).
- AT × Group is also negative (*B* = −.241, *p* < .001). The teacher slope≈ +0.037 (marginal), while the student slope ≈ −.204. Thus, a stronger attitude relates to weaker gains in EF for students, but little reduction for teachers (AT x Group of Fig 1), indicating that the facilitative effect of AT on EF is stronger on the teacher side.
- AB × Group is positive (*B* = +.120, *p* = .002). The teacher slope≈ +.185, and the student slope ≈ +.305, showing that AB more strongly promote EF among students (see AB x Group of Fig 1).
- PR × Group is not significant (*B* = 0.053, *p* = .22) (PR x Group of Fig 1).

In sum, relative to students, teachers showed a more favorable association of EF with EX and AT, whereas students showed a stronger association of EF with AB; no between-group difference emerged for PR.

#### 3.3.3 Institutional policy and group interaction.

**Fig 2:**
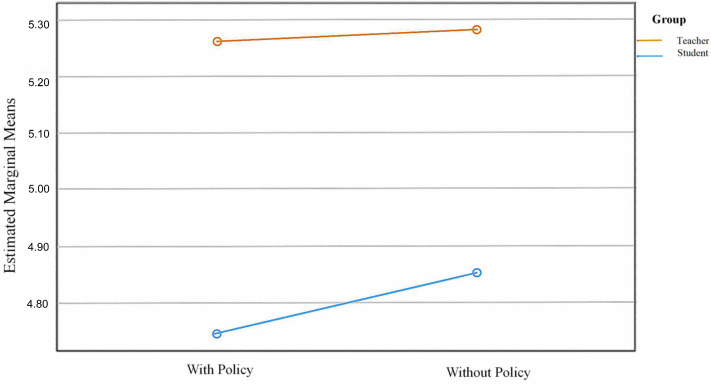
Estimated marginal means of EF by institutional AI policy and group. Estimated marginal means of EF conditional on the presence of an institutional AI-use policy (“With Policy” vs. “Without Policy”), plotted separately for teachers and students. Lines depict the Group × Policy interaction tested in Model 3, which was not statistically significant.

Building on Model 2 by adding UR × Group, Model 3 did not improve in explanatory power ($\Delta R^{2}$ = .000, *f*^2^ = .000, no incremental explanatory gain. From the adjusted means plot (Fig 2), the teacher group’s EF evaluations were essentially unchanged between policy vs. no policy schools (5.28 → 5.29), whereas the student group showed a slight increase in EF evaluations in universities without a policy (4.73 → 4.86). The pattern is consistent with the earlier “net effect” description: the presence of a school policy does not significantly moderate Group, and the UR × Group interaction adds no additional explanatory power.

#### 3.3.4 Institutional tier × group interactions.

After additionally entering the three interaction terms UT 2 × GrC, UT 3 × GrC, UT 4 × GrC, Model 4 showed a clear improvement in fit (*R*^2^ = .480, adjusted *R*^2^ = .467). The institutional tier × Group block in Model 4 provided a small incremental improvement in fit (*f*^2^ = .067). The difference is larger for first-class institutions, with *B* = .986, *p* < .001. In short, institutional hierarchy amplifies teacher–student differences, most prominently in Double First-Class universities.

**Fig 3:**
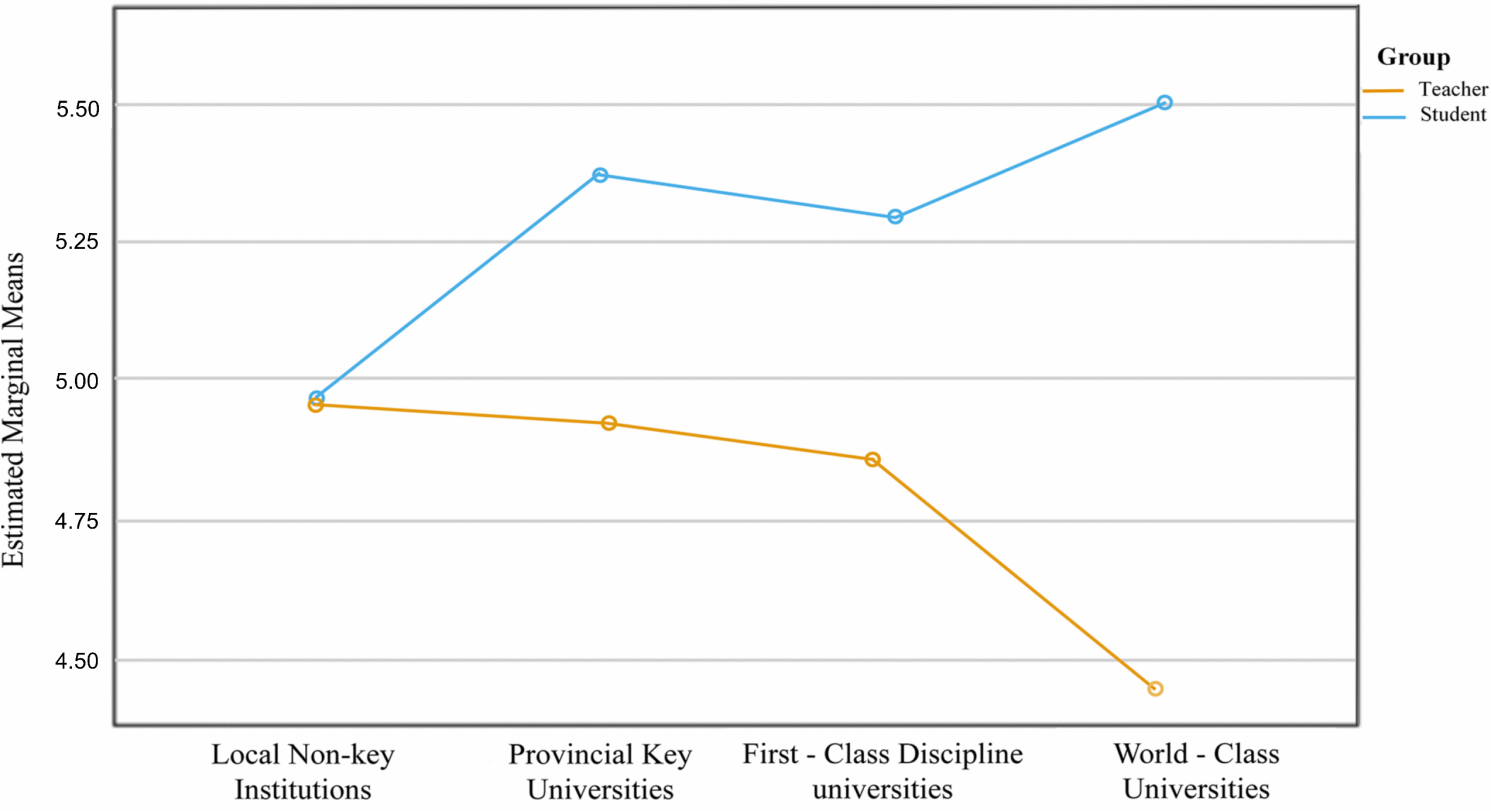
Estimated marginal means of EF by university type and group. Estimated marginal means of EF (adjusted for covariates) across four university types, plotted separately for teachers and students. The plot visualizes the University Type × Group interaction from Model 4: teachers consistently report higher EF than students, and the teacher–student gap widens with institutional tier, reaching its maximum at Double First-Class universities.

This pattern is evident in the adjusted means plot (Fig 3). Holding EX, AB, RU, and AT at their means (z = 0), in ordinary undergraduate institutions the estimated means of EF for teachers and students are close (teachers ≈ 5.00, students ≈ 4.98), with a small gap. Moving to provincial key and First-Class Discipline universities, teachers’ EF evaluations remain elevated (5.35 → 5.28; minor fluctuation but overall high), while student evaluations decline slightly (4.93 → 4.86), and the teacher–student gap begins to widen. In Double First-Class universities the divergence is largest: teachers’ estimated EF rises further (5.49), whereas students’ estimated EF drops markedly (4.45), consistent with the strong positive effect of UT4×GrC in Table 5 (*B* ≈ .986).

#### 3.3.5 Disciplinary category and group interactions.

With DT variables added, Model 5 further improved in fit (*R* = .714, *R*^2^ = .510, Adj. *R*^2^ = .494), indicating that the final model explains 51.0% of the variance in the dependent variable. The disciplinary block further improved model fit with a small incremental effect (*f*^2^ = .061). The standard error of the estimate decreased to .456, further enhancing model fit. The results show that disciplinary category × Group interactions make a substantive contribution to the dependent variable.

**Fig 4:**
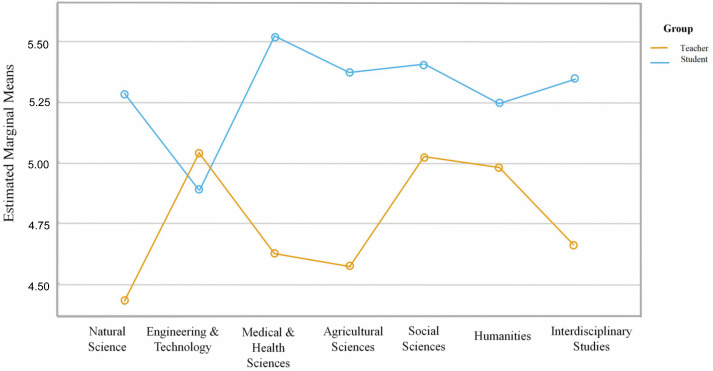
Estimated marginal means of EF by discipline and group. Estimated marginal means of EF across seven OECD-aligned disciplines, plotted separately for teachers and students. Values are adjusted for EX, AB, RU, and AT (all z-standardized), set at their means. Lines visualize the Discipline × Group interactions from Model 5: the teacher–student gap is largest in Natural Sciences and narrower in Engineering & Technology, Social Sciences, and Humanities.

The estimated marginal means plot (Fig 4) reveals a clear pattern: the teacher–student gap is largest in Natural Sciences (teachers ≈ 5.27, students≈ 4.45), corresponding to the reference group and a larger baseline gap. In Engineering & Technology, Social Sciences, and Humanities, teachers’ evaluations remain higher than students’, but the gap is notably smaller than in Natural Sciences (teachers ≈ 5.2 – 5.3, students ≈ 4.95 – 5.05), consistent with the significant negative coefficients for DT2×Group, DT5×Group, and DT6×Group. For Medical & Health Sciences, Agricultural Sciences, and Interdisciplinary Studies, the separation of the curves does not change significantly relative to Natural Sciences, aligning with the non-significant interaction terms.

## 4 Discussion

### 4.1 Overview of findings

Overall, the findings indicate five key patterns: (1) AI participation and perceived ability gains exhibit stable positive main effects on evaluations of perceived thesis quality, and an explicit institutional policy also shows a positive main effect; (2) the moderating effect of Group is significant, teachers place greater emphasis on the extent of students’ use and their attitudes toward use, whereas students perceive more salient gains in ability; (3) policy presence does not significantly moderate teacher–student differences; (4) institutional-tier and Group interactions indicate that teacher–student divergence is largest in Double First-Class universities and smallest in ordinary institutions; and (5) disciplinary-category and Group interactions indicate the largest gap in Natural Sciences and comparatively smaller gaps in Social Sciences and Humanities.

### 4.2 Individual level

The positive main effects of EX and AB align with the “perceived usefulness → performance” linkage in technology acceptance research [41]. On the student side, the moderation of AB is stronger, indicating that students are more sensitive to the “visible gains” brought by generative tools—improvements in language expression and structural optimization—and thus are more likely to believe that AI has enhanced their abilities. By contrast, the effect is weaker for teachers, who are less convinced that AI use reflects genuine capability gains or authentic learning [8]. Teachers place stronger emphasis on authentic learning—that is, on students genuinely acquiring knowledge and capabilities that underpin long-term growth and development. From this standpoint, GenAI is only one tool among many; the key is learning how to use the tool well rather than focusing merely on its immediate outputs [42,43], On the teacher side, the stronger moderation of AT reflects greater attention to academic norms, evaluation standards, and how to integrate GenAI into writing workflows in a compliant manner, rather than focusing solely on end products.

However, PR remains positively associated with EF after controlling for other variables, which may suggest that risk awareness coexists with metacognition. Identifying and checking potential problems can encourage more cautious use and, in turn, higher-quality outputs [24,44].

### 4.3 Organizational level

Although UR is significant, its main effect is weak, suggesting that policy presence may operate more as a baseline governance signal than as a strong driver of evaluative judgments [15]. The non-significant UR × Group interaction further indicates that policy presence does not differentially shape teacher versus student evaluations, consistent with the view that formal guidelines set minimum compliance expectations but vary in implementation intensity across settings. By contrast, the significant UT × Group pattern—most pronounced in World-Class universities—can be interpreted through differences in evaluation regimes and institutional missions. In higher-tier research universities, thesis evaluation tends to emphasize originality, methodological traceability, and evidence of independent scholarly contribution; under such standards, GenAI assistance may be scrutinized more closely, contributing to stronger teacher–student divergence in perceived thesis-quality evaluations [8]. In ordinary institutions, where assessment may place relatively greater weight on linguistic clarity and overall presentation, GenAI-supported improvements in expression and structure may be more readily recognized in evaluations [25]. Taken together, these tier-related differences suggest that institutional hierarchy shapes how GenAI use is interpreted against locally salient quality criteria, thereby amplifying role-based divergence in EF

### 4.4 Disciplinary paradigms of GenAI use

Disciplinary differences can be theorized in terms of epistemic norms and evidentiary demands—namely, what counts as a credible contribution and what kinds of evidence are required to justify claims and authorship [45]. In the natural sciences, where rigorous empiricism and reproducibility are central, the boundaries of GenAI use tend to be more sensitive; concerns about data provenance, code reproducibility, and the attribution of ideas mean that perceived thesis-quality gains may be discounted when outputs cannot be traced to verifiable procedures (Nature Machine Intelligence Editorial, 2023). In engineering, where work is often organized around toolchains and process validation, GenAI can be more readily incorporated into structured workflows, although standards and review gates still delimit acceptable use [25]. In the social sciences and humanities, where argumentation, synthesis, and clarity of expression are salient criteria, GenAI-supported improvements in drafting and rewriting may more readily translate into perceived quality gains, with guardrails focusing on source verification, citation accuracy, and voice consistency [23]. Across fields, transparent disclosure of GenAI assistance is likely to be important for aligning perceived benefits with discipline and specific evaluation norms

### 4.5 Research contributions and practical implications

Building on prior work that often reports efficiency and quality gains from GenAI in academic writing, this study advances boundary understanding in two ways. First, it identifies who benefits more: on the teacher side, stronger normative attitudes and tighter control of participation translate more readily into high-quality outputs, whereas on the student side, perceived ability gains translate more into positive evaluations. Second, it identifies where effects are stronger: teacher–student differences widen as institutional tier rises and as the natural-science paradigm becomes more prominent. In this way, we situate technological effects within a three-dimensional context of identity–institution–disciplinary paradigm, adding contextualized evidence on the educational use of GenAI. The effect of GenAI on perceived thesis quality is not homogeneous; it depends on individual-level perceived ability and normative attitude, organizational rules and tiers, and the practical logic of disciplinary paradigms.

Educational governance may benefit from shifting the focus from “whether to use GenAI” to “how to integrate GenAI with compliance and learning goals.” For teachers, a feasible approach is to provide lightweight, practice-oriented support on disclosure, verification, and process-based assessment of GenAI-assisted writing, so that expectations can be applied consistently without substantially increasing supervisory workload. For students, capability-oriented guidance is likely to be most implementable when packaged as a minimal, teachable workflow—prompting → verification → reflective revision—paired with simple evidence requirements that make learning gains more observable. At the institutional level, differentiated norms may be more viable if introduced in a staged manner and aligned with existing thesis-management procedures, rather than adding parallel compliance systems

## 5 Conclusions and limitations

### 5.1 Conclusions

Drawing on student–teacher dual perspectives, this study evaluates the impact of generative AI on the quality of undergraduate theses. The findings show that, in the current Chinese context, both the degree of GenAI participation and perceived ability gains are stably associated with higher quality evaluations, while explicit institutional policies are generally beneficial but modest in effect. In terms of pathways, teachers rely more on the positive coupling of “norms and participation,” whereas students are more likely to translate “ability gains” into perceived quality. Institutional tier and disciplinary paradigm serve as important boundary conditions—teacher–student differences are larger in high-tier institutions and natural-science contexts, and smaller in engineering, social sciences, and humanities; the interaction between “policy presence” and identity is not supported. Accordingly, we recommend translating high-level norms into operational procedures and verification checklists, building a student training pathway of “AI draft → personal rewriting → evidence strengthening → reflective revision,” and providing contextualized guidance and evaluation tailored to institutional tier and disciplinary characteristics.

### 5.2 Limitations and future research

This study’s sample is concentrated in specific regions and institutional types; it includes only general undergraduate institutions and does not cover vocational undergraduate programs, despite disciplinary particularities. Methodologically, the study relies on a one-shot cross-sectional survey and uses hierarchical regression to test main and interaction effects, supporting correlational rather than causal claims; interactions are limited to second-order terms, without testing higher-order interactions or nonlinearity. Future research could expand across regions/countries and a broader range of institutional tiers and types to enable cross-cultural and cross-regional comparisons. Methodologically, future work may adopt multilevel models and further examine mediating linkages. EF and AB reflect perceived (not objectively verified) thesis quality and ability development, and PR reflects perceived risks/problems, which may also capture role-based standards or familiarity with GenAI. Future work should incorporate longitudinal designs and objective quality/process measures to test competing explanations.
